# Supplementary material for: Understanding climate refugees from educators’ perspectives: Social studies teachers’ views
Source: PLoS One. 2026 Mar 16;21(3):e0344777. doi: 10.1371/journal.pone.0344777 (PMC12991243; doi:10.1371/journal.pone.0344777)
Supplement: S1 File — (PDF) [file pone.0344777.s001.pdf]

PARTIPICANT 1(K1)
Now, briefly about refugees—I hope I can find it from somewhere. One moment, that's
also there. Let me save it, let me save it. I sent the file, but I didn't open it from here.
Let's log in. Yes. Brother, climate refugees briefly—I mean, I didn't define it there, but
here generally I need to give some detail. Here, people generally think of climate refugees as people who migrate due to climate in a minor way, but it's necessary to make
a distinction between *refugee* and *migrant*. A refugee means a person who migrates
forcibly. Climate refugees are also people who migrate forcibly. So they are not migrants.
Migrants are individuals who migrate to live in a more comfortable area or a freer environment, whereas refugees are individuals who are in much harsher conditions.
That's why it needs to be distinguished. And now there is such a population group in the
world. I just wanted to give that information first, brother. Let me save something, let me
check it instantly. Brother, how many years has your service period been?
20.
Mashallah, that's great. Your field is based on Social Studies, right brother?
Yes.
The school type—are you currently working in a regular school, brother?
Middle school.
Ah, middle school. So, do students or families have an inclination toward environmental-
type topics like this? Do they have any prior knowledge? Does it affect them? Or not?
Have you ever encountered a climate refugee before, brother? Do you have such an
experience?
No.
But here, of course, we should think internationally, right?
Domestic could also be possible actually. Individuals in the Konya region could also be
examples.
If it's domestic, for example, a large dam was built here. Some villages had to be relocated. People lost their land. A new living area was established, houses were built for

them, etc. So they experienced a small-scale relocation. But as a result, their villages
disappeared. All their memories, homes, lands—everything was gone. Forced.That's something that happens frequently in Turkey anyway due to dams.
Exactly.
But this dam issue is a bit political, brother. I mean, it's not actually climate-related, but it
could have a connection like this: if the dam was built to transfer water to other regions
of Konya or Karaman, for example, and when drought becomes active in those regions,
then it becomes climate-related. But in Turkey, generally, there is respect for people and
history in this context. They don't usually just build a dam and move on like that.
Exactly.
We've had a lot of that in our hometown too. So that's why. According to you, what does
a climate refugee mean, brother, if you were to define it?
Well, in my opinion, a climate refugee is when people are forced to leave the places
where they live due to severe natural events. Climate refugees are people who have to
move because of drought, extreme cold, excessive rainfall, and the frequent occurrence of
floods related to these, which make life unfavorable, and people who want to escape from
this are forced to move to different regions or geographies.
Alright, brother. Have you ever received any training or seminar on climate refugees
before? Or have you seen it in the news?
No, I haven't.
Alright.
We see some small things on social media, but…
I mean, there isn't a complete definition.
Yes, I haven't received any special training, seminar, or course yet. And it's not
something that is very much on the agenda in Turkey at the moment.
Do you think there is a difference between climate refugees and other refugees?
There is.
There is in this way: other refugees' situations are more human-centered—wars,
conflicts, etc. These things occur even in superpower countries like the United States or
in different regions. Normal refugees are mostly groups that emerge as a result of wars.

Recently, we have observed globally that refugees frequently migrated due to events like
the Syrian conflict, the Afghan wars, and terrorism-related issues. But these are
preventable things—wars, for example, can be resolved through international dialogue
and such. But climate is different.Who do you think is responsible here, brother?
If we were to distribute responsibility for
the emergence of climate refugees, who bears the most responsibility?
Are you asking in terms of climate refugees?
I mean, who are the individuals responsible for the emergence of climate refugees?
.
But reducing this.
States don't want to give this up, of course. Production would decrease, and they wouldn't be able to sell goods to the world or produce enough goods for their own country's needs. They think this would disrupt their economic balance, so they oppose it.
So state policies are extremely important.
This will reduce global warming. Climate crises worldwide already occur due to global
warming. Floods are the same, drought as well. Extreme and irregular rainfall, etc. All of
these fundamentally stem from global warming. And at the root of global warming is the
fact that we are increasingly poisoning the world with carbon emissions—collectively, as
countries and as the world. Unfortunately, we have disrupted the world's balance. That's
why climates are completely mixed up. Rainfall that should occur in spring has shifted to
June. Winter that should come in November or December has shifted to February or
March. The familiar climate order has changed. For example, speaking for this region: we
should have abundant rainfall in April and May, but it didn't rain at all during that period.
Now it has just started to rain a little, but in the form of sudden downpours. It doesn't
benefit the soil much; sometimes hail suddenly falls and agricultural lands are destroyed,
etc. In short, people's lifestyles and state policies are the factors that trigger this climate
crisis and, consequently, climate refugees.

It is said that 1 billion people will be affected within the next 50 years. What do you think
about this? There is such an assumption by UNESCO.
Yes, that's true. I largely agree with that. If UNESCO has made such a projection, it must
be based on scientific infrastructure. When we look at our current environment, right now
the Ermenek Dam is right in front of me. It's a massive dam, but it can't generate electricity because the water level is insufficient. Because it didn't snow at all this year in
Ermenek city center. It snowed a little in the highland areas.
In our village, some lands have completely dried up.
In the past, I could irrigate my field in 15 minutes; now I could barely irrigate it in 1.5
hours last year. So that figure of 1 billion—maybe it could change by plus or minus 200
million—but it's still a very accurate projection. Unfortunately, if this continues, with the
capitalist world's mindset of earning more, producing more, selling more, living more
luxuriously—we humans have become very comfort-oriented. Now there is almost onecar per person in every household, as many motorcycles as people, and so on. Everyone
wants to travel more comfortably, with less effort or no effort at all. Of course, this will
further intensify the climate crisis. As a result, climate problems will increasingly affect
human life deeply. This will leave people desperate and force them to seek refuge elsewhere, domestically or internationally.
Those who relocate within the country may have it somewhat easier, but those who are
forced to migrate abroad will be in a much more difficult situation. Even though it is a
great necessity, almost no country will welcome them. Those countries will also be
experiencing climate crises. Even if not as severe, their situation won't be very good
either. So countries will say, "We can barely feed ourselves, and now we have to feed
climate refugees?" Just as some groups in our country opposed Syrians in the past, saying
"We can't even find jobs ourselves, are we supposed to find jobs for them?", other

countries will also not want them. Even if people manage to go, they will live under very difficult conditions—second-class, marginalized, confined to certain areas. They will feel different in many ways.

So this refugee crisis will be a problem for the entire world, both for receiving countries and for countries people are forced to leave. Especially countries with insufficient rainfall, countries experiencing sudden floods, and coastal regions threatened by rising sea levels will be more affected. People living in these areas will be forced to migrate just to survive. Just as people migrate today due to war, in the future they will migrate due to drought, hunger, and related problems, knocking on the borders of other countries, trying to enter legally or illegally.

Do you think people's attitudes toward climate refugees would differ from their attitudes toward other refugees?

Yes, I think it would. For example, when people migrate due to war, some might say, "You should have stayed and defended your country." But climate is beyond human control. People may think that climate refugees couldn't prevent what happened to them, so they might be viewed more sympathetically. Someone coming to France due to constant floods might be treated more humanely. Climate refugees are more innocent in that sense, and acceptance rates may be higher. Governments may be more generous, and local people more welcoming.

Do you think climate refugees should be granted citizenship?

Yes, it can be granted, and even should be. Because the possibility of their regions returning to former conditions is very low. Where would they return to? How would they survive in a desertified area? What would they farm? Where would they graze animals? Nothing. In contrast, people who flee wars may return when conditions improve. We saw this with Syria. But climate crisis is irreversible. So a different legal status should be

created for climate refugees.
Should the topic of climate refugees be included in the social studies curriculum?Definitely. It is already covered indirectly—global warming, natural disasters,
migration—but this topic could be presented under a specific heading. It would raise
awareness and help students understand how climate refugees emerge and encourage
respect for nature.
Have you ever taught this concept in your classes?
We teach it as forced migration due to climate conditions, but using the term "climate
refugee" might be more effective.
Which methods and materials should be used?
As a social studies teacher with 20 years of experience, I would mainly use the case study
method. Examples from Turkey and the world—small-scale or international migrations—
should be analyzed with students, allowing them to derive conclusions themselves.
How should education be provided to climate refugee individuals?
First, language education is essential, then cultural integration. Education should involve
not only students but families as well. They should also be taught environmental sensitivity. Over time, children adapt through school and social life.
What can be done to raise awareness?
Students should ask themselves daily: "How should I live today so that I or future generations don't become climate refugees?" A frugal, environmentally respectful
lifestyle should be promoted. Activities by NGOs, tree planting, analyzing why these
actions matter—all should be discussed.
I'm currently an assistant principal. We constantly remind students to turn off lights, save
water, not litter. Some students now do these things instinctively. They even report
broken faucets. When students feel they contribute to the future, they feel meaningful.
When people realize they are part of nature, they protect it. Meaning creates value. Otherwise, behaviors are not sustainable. Understanding the "why" is crucial.
Is there anything you would like to add?
I think the concept of climate refugees should explicitly enter the social studies

curriculum and textbooks. We already teach it implicitly, but making it explicit would be
better. The refugee concept creates awareness of negative outcomes and encourages
environmentally responsible behavior. This could help reduce the projected 1 billion
climate refugees. This study will present such recommendations to curriculum developers.
Thank you, brother.Thank you.
Let's stay in touch if needed. I'll stop the recording now if you have nothing to add.
Okay.
Thank you very much.
PARTIPICANT 2 (K2)
So, sociologically speaking—can you say that this is a region with environmental sensitivity, for example?
The area where my school is located is one that I would describe as socioeconomically
disadvantaged. Students' income levels are low. Interest in education is low. It's that kind
of area. But it's also a mixed area. A cosmopolitan area. All kinds of students come.
Students are diverse as well. Have you ever encountered a climate refugee before?
As you mentioned earlier, it is an environmentally driven forced migration. Would a
flood count as that?
No. It's a natural disaster, but a different type of natural disaster.
For example, some of my students' families used to do agriculture in villages. When their
income was not sufficient, they migrated to the city. It may seem economic, but if we
look at the root cause, it is the insufficiency of agriculture.
We need to look at the nature of that insufficiency—whether it is related to climate,
whether it is related to drought. If I come across such a case, I would like to examine it.
Honestly, I would like to work with such groups. In this context, based on what we have
discussed, how would you define a climate refugee in a striking way? What does it mean
to you?
As we discussed earlier, if I think of the difference between a climate refugee and normal

migration: normal migration is mostly done for economic reasons, to reach better opportunities, whereas climate refugees are more related to necessity—this could be
drought, it could be flooding, again drought or flooding.
Alright. Have you ever received any training or seminars about climate refugees before?
No, I haven't.
Can you explain the difference between climate refugees and other refugees? We explained refugee versus migrant, but what differences do you think exist between other
refugees and climate refugees?When I think of the concept of a refugee, escaping from war comes to mind directly.
Climate refugees, on the other hand, are escaping due to environmental problems.
Alright. What do you think about climate change causing forced migration, especially
within the next 50 years? It is said that there is a possibility that it will negatively affect
people's lives and push them into refugee status. This is among UNESCO and UNEP
data—more precisely, their projections. What do you think about this?
If I think in terms of my country, I don't think our country is among the most disadvantaged regions climatically. But there are countries that are much worse off than
us. If things get even worse for them in the next 50 years, it seems like countries will
have to deal much more with migration and refugee issues politically.
Alright. Do you think climate refugees can be considered a vulnerable group? Can we
count them as a victimized group?
Yes, we can. For example, a man has a house, has land, then a flood comes—his house is
gone, his land is gone. He is a victim.
Exactly, a victim. Who do you think are the causes of this victimization, or who are
responsible for the emergence of climate refugees? Who can we hold accountable?
It depends on the precautions taken. If you build your house on a riverbed, then you are
partly responsible. The administration that allowed it may also be responsible.
But don't think only in terms of riverbeds. Think in terms of drought or sea-level rise.
Think of island countries.
If we talk about sea-level rise, there is this common idea that countries like the

Netherlands will be submerged in a few years.

Apart from the Netherlands, in some small islands right now, and in regions like India, there are migrations occurring due to sea-level rise. That's what I mean. We haven't reached the Netherlands yet. We will get there. The Netherlands is like a palace, my brother. They can take precautions themselves.

Then there is nothing people can do in that case. It's something natural.

Then we need to look at the cause of climate. Where are we? How do you evaluate climate refugees from a social or political perspective? As a society, would we accept them, or do you think they should be accepted?

First of all, I think this is a very new concept. I mean, I'm a teacher, and this is the first time I've heard this term.

Exactly.I think society needs to become aware of this issue.

Let me also mention this: currently, according to the data, out of 84 million refugees, 9.8 million are recorded as climate refugees.

About 10–12%.

Exactly. That's not a small proportion. And internationally, this issue has been discussed for quite some time, but it is not fully defined as "refugee" by organizations such as the United Nations. Because if it is defined as such, then other countries would have to accept asylum seekers. That's why there are problems with definition. It doesn't fully settle on a legal ground. They relate it to the Geneva Convention and similar frameworks.

So it is not fully formalized. But the reality exists. Whether it is legally defined or not does not change the fact that it exists. And by the way, many climate-induced migrations within countries are not recorded. The 9.8 million refers to those that are recorded.

Is that figure for Turkey?

No, for the world. Out of 84 million migrants. These are annual figures—migrations that occurred in one year worldwide, specifically in 2024.

Is it clear which countries are most affected?

Mostly South Asia and African countries.

Exactly.

Actually, another problematic region right now is the region where Turkey is located—
Turkey and Pakistan. So we are within a risky zone. Because the Middle East, the hot
belt, Africa, and its continuation are problematic. In fact, Africa is connected to South
Asia by land. Pakistan is connected to India and Bangladesh by land. It is easier to
migrate by land than by sea. You know these dynamics. So the migrants coming from
there make this region risky.
Are we in the group that will receive migration, or the group that will send migration?
We are both. And we are a bridge. That's my current view. Since we are much more of a
transit zone than the current migration numbers suggest.
So it affects us as well.
That's why this is a very important topic. It needs to be addressed—not only
educationally. There has been almost no study in education so far. I'm trying to gain a
foothold in the educational dimension, which could produce serious results. It's a term
that most people don't know. I didn't know it either until I became interested. I
encountered it while reading my thesis literature. Otherwise, as I said, most people
don'tknow about it. But this reality is gradually coming to light, and there needs to be an
answer to the question of whether these individuals should be accepted as citizens or not.
It seems like there are many legal loopholes. Someone could use this as a pretext to seek
asylum with bad intentions. People who couldn't migrate for other reasons might use this
excuse.
That's why they don't recognize them as refugees. But there is another side to this, which
I will explain later. I don't want to direct the conversation right now. I shouldn't
influence the data. Do you think climate refugees should be included in the social studies
curriculum? Should it be taught?
I think it should be included. Because with the new education model this year, especially
in fifth grade, there are many new concepts. We teach new concepts to children. There

are many newspaper-like texts that caught my attention this year. We explain topics
through these news items. If there were news related to this, we could discuss it with
children.
You said you hadn’t taught it before. Do you think this concept should be included as a
separate heading? Not just implicitly, but explicitly?
I didn’t teach it—well, I did teach it, but I didn’t know it by the name “Climate Refugee.”
I taught natural-resource-based migration and economically based migration within
general migration. But the term “Climate Refugee” could be included explicitly in
textbooks.
Exactly. So you think it should be included. In teaching this topic, which methods and
techniques should be used? With which methods would we get better results?
Since it is a new concept, brainstorming could be used. A brief introduction could be
given first, then students could be asked what they think about it, and afterwards the main
information could be presented.
As a social studies teacher, if you take responsibility, what can be done to bring society
together on this issue? You said society needs awareness—what can be done?
From a school perspective, videos on smart boards or future-oriented simulations could
be used. If migration happens like this, this will happen; if you go, you will go like this;
there will be this much migration. Then, what problems might arise, what solutions might
exist—these can be addressed through simulation methods.
You mean to make it more realistic, to make them feel it. When climate refugee
individuals arrive, what kind of education should be provided to them? When they
become part of this society, how should their education be handled? Do you have any
ideas?If a refugee student knows the language, teachers usually try not to separate them and
teach the same lesson as others. Climate refugees might be more sensitive about
environmental issues. They could be given more opportunities to speak in class. For

example, when teaching disasters, that child has experienced it—they could share their
experiences. That's how support could be provided.
Alright. Is there anything else you would like to add? Thank you very much. You can
stop the recording now.
PARTICIPANT 3 (K3)
Social Studies Teachers' Views on Climate Refugees
This interview form was prepared to understand social studies teachers' knowledge,
attitudes, and educational practices regarding climate refugees. Your responses will be
kept confidential and used solely for scientific purposes.
A **climate refugee** is a person who is forced to leave their place of residence due to the
adverse effects of climate change. These individuals migrate in search of a safe living
environment in the face of environmental threats such as floods, droughts, desertification,
and sea level rise. Although they are not legally recognized as "refugees," their forced
displacement reveals the humanitarian dimension of the climate crisis.
Gender: Male
Years of Service: 2 years
Field of Graduation: Social Studies Teaching
School Type: Middle School
1. Have you ever encountered a climate refugee before? What do you think the term "climate
refugee" means?
I have never encountered one. I think it refers to a concept related to climate. Since the
term includes "refugee," it can be said to mean a person who migrates forcibly.
2. Have you ever seen any education, seminar, or news related to climate refugees? Please
explain briefly.
No, I have not.3. Is there a difference between climate refugees and other refugees? If so, what are they?
In my opinion, they are similar in terms of the events they are exposed to and their consequences. However, the severity may differ. For example, we cannot put war refugees and climate refugees in the same category.
**4. What do you think about climate change causing forced migration?
Follow-up: It is assumed that one billion people will become climate refugees within the

next 50 years. What are your thoughts on this?**
I evaluate all migrations that occur against our will as negative. In the coming years, an
increase in this situation may lead to crises.
**5. Do you think climate refugees can be considered a disadvantaged group? Why?
Follow-up: Who do you think is responsible for the increase in the number of climate
refugees?**
They can be considered disadvantaged because the main cause of climate change is
human activity. As a result, some people become victims due to these reasons.
**6. How do you evaluate the social or political perspective toward climate refugees?
Follow-up: Should climate refugees be granted citizenship?**
The same thing could happen to us as well. If it is carried out in a controlled manner, I do
not see any negative situation. Of course, the whole world should take responsibility.
7. Do you think the topic of climate refugees should be included in the social studies
curriculum? Why?
Of course, it should be included, especially in social studies classes. This is because
social studies is a course that explains sciences related to society and teaches students
how to apply this knowledge in their daily lives.
8. Have you ever addressed this topic in your classes? If so, what method did you use?
I have not addressed it.
9. Which methods, materials, or activities do you think would be effective in teaching this
topic?
It can be explained using concrete examples. It can be demonstrated to students through
local examples. Videos and slides can help make the topic more concrete.10. As a social studies teacher, what can be done to increase awareness on this issue?
Presentations on climate change can be prepared. Events that cause climate change can be
examined, and efforts can be made to prevent these situations. Brainstorming can be used
in the classroom to involve students actively. In this way, they will internalize the topic.

11. What methods should be used when providing education to individuals who are climate
refugees?
Practices and educational programs that help integrate them into society should be
implemented. Psychological and sociological education is especially important. They
should be told that the situation they are in is normal. Important knowledge and skills
should be transferred to help them become part of society.
12. Do you have any additional opinions or suggestions?
We need to become aware before this situation happens to us. This is only possible through education. Therefore, educators should be trained first, and then they should
transfer what they have learned to students.
PARTIPICANT 4 (K4)
nterviewer: First of all, welcome.
Participant: Thank you.
Interviewer: How are you?
Participant: I'm fine, thank you.
Interviewer: How about you?
Interviewer: I'm fine as well. Yes, we had previously planned to conduct an interview
with you.
Participant: Yes.
Interviewer: It is about climate refugees.
Participant: Yes.
Interviewer: First of all, your gender is male.
Participant: Yes.
Interviewer: How many years have you been working?
Participant: Twelve years.**Interviewer:** You have been working for twelve years. What is your field of graduation?
Participant: Social Studies Education.
Interviewer: Social Studies Teaching, okay. And the type of school you currently work
at is a public middle school.
Participant: Yes.
Interviewer: Okay. Now we are moving on to the questions.
Our first question is: Have you ever encountered a climate refugee before? What do you
think the term "climate refugee" means?
Participant: Thank you. I have never encountered a climate refugee before. Although I

have heard this concept many times, I have never personally met a climate refugee. In my opinion, a climate refugee refers to people who become unable to live in their own country due to disasters caused by climate-related factors. These people are forced to migrate to another country—or rather, to other countries. I want to emphasize the word *forced*. Climate refugees are people who are compelled to migrate. They are forced to leave their own country and migrate to other countries, just like war refugees. They have to escape because in both cases there is a vital, life-threatening problem.

Interviewer: Our second question is: Have you ever seen any education, seminar, or news related to climate refugees? Could you briefly explain?

Participant: There are educational contents about climate refugees in the educational sections of EBA. There are also trainings related to climate crises. I have seen such educational materials before. I have also encountered related content in national and international media. However, so far, most of the focus has been on the climate crisis itself. Climate crises are emphasized much more, but there are not many major news reports yet about the large-scale migration that will follow. At the moment, the main focus is on the climate crisis.

Interviewer: Have you taken a direct course or seminar on this topic?

Participant: No, I have not taken a direct course or seminar.

Interviewer: I see. Our third question is: Is there a difference between climate refugees and other refugees? If so, what are they?

Participant: There is not much difference between climate refugees and other refugees, because both are people who are forced to leave their countries due to threats to their lives. However, if we look at the differences, wars can occur in relatively limited geographical areas, whereas climate crises will spread over much larger areas. Therefore, I predict that the number of climate refugees will be much, much higher than the numberof war refugees. I also think that this situation concerns Turkey greatly, since Turkey is

located on major migration routes.

Interviewer: Our fourth question is: What do you think about climate change causing forced migration? It is assumed that one billion people will become climate refugees within the next fifty years. What are your thoughts on this?

Participant: As I mentioned earlier, climate refugees cause forced migration. People are compelled to leave their countries due to life-threatening consequences caused by climate crises. I think that the actual number may even exceed the estimated figures. As I said before, Turkey, being located on migration routes, will be one of the countries most affected by this situation. I believe that in the future, countries that are self-sufficient will become more important, and migration will be directed toward such countries. I think Turkey will be one of the countries receiving the most migration because it has water resources and a structure capable of coping with climate change, especially in the Eastern Anatolia region. Therefore, I believe that Turkey will receive the highest level of migration, particularly toward the Eastern Anatolia region, since we live in a country located on major migration routes.

Interviewer: Another question asks: Do you think climate refugees can be considered a disadvantaged group? Why? And who do you think is responsible for the increase in the number of climate refugees?

Participant: Yes, I think climate refugees can be considered a disadvantaged group. Just as people are considered victims in wars, and war refugees are regarded as disadvantaged, climate refugees should also be evaluated in the same way. The logic is the same. People are forced to leave their countries because their lives are in danger. Climate refugees are also forced to leave their countries because their lives are at risk. Hunger threatens life, drought threatens life. Therefore, climate refugees can definitely be considered a disadvantaged group.

As for who is responsible for the increase in their numbers, the greatest responsibility lies
with major industrialized countries. I believe that countries with advanced industrial
development are the main contributors to the climate crisis. Industrial pollution, soil
contamination, and severe air pollution have caused changes in the climate. These changes have led to drought and, ultimately, to the problem of climate refugees. Therefore, responsibility clearly lies with those actors.

PARTIPICANT 5 **(K5)**

Individuals we refer to as climate refugees are those who are forced to migrate from their
region due to climate-related factors. In other words, this is not a choice-based migration
but a forced one, and it is caused by climate conditions. There is now such a type of
migrant in the world. Internationally, they are not defined under the category of "refugee," but they can be considered under the category of "migrant." They are not
officially recognized as refugees because recognizing them as such would create serious
challenges, particularly for wealthy countries. Therefore, this concept has not yet been
formally recognized by the United Nations. However, studies on climate refugees do
exist in the literature, and they are quite extensive and increasing every day. That is why I
wanted to examine this issue in education, where no studies have yet been conducted.

These individuals migrate due to climate-related factors such as drought, floods, and sea-
level rise.

After defining this concept, may I ask how many years you have been working, sir?

Participant:

Thirty years.

Interviewer:

Thirty years, impressive. What is your field of graduation?

Participant:

History. Istanbul University, Faculty of Letters, General History.

Interviewer:

You graduated from the History Department. Later, you transitioned into Social Studies

teaching.

Participant:

We transitioned out of necessity. Under the conditions of that time, the Ministry reassigned us to Social Studies.

Interviewer:

Was that around 2006–2007?

Participant:

Yes, around 2006–2007.

Interviewer:

Had you encountered the concept of climate refugees before?

Participant:

No, we had not encountered them. Perhaps they existed, but we were not aware of them.

When we talk about climate-related migration, there are historical examples. As a historian, I find this interesting. Throughout history, droughts have caused the collapse oremergence of civilizations. Migration has always existed. However, today we can scientifically identify where people migrate from and why. So, defining this phenomenon makes sense to me.

Countries do not legally accept this classification anyway. Since it has not fully entered legal literature, states do not recognize it in their laws. There may be fears behind this.

Once it is defined, millions of people affected by global climate change may migrate north or south, which would create political problems. That may be why states avoid recognizing it.

Historically, climate has always shaped civilizations. When water sources dry up, even the greatest civilizations collapse. Many civilizations disappeared due to climate-related reasons. This is not a new phenomenon; it simply was not defined before.

Interviewer:

refugees?

Have you ever received training or attended a seminar specifically about climate

Participant:

I am aware of climate change issues, but I do not recall any striking seminar specifically about climate refugees.

Interviewer:

Do you think there is a difference between climate refugees and other refugees?
Participant:
Other refugees may flee due to war, political regimes, or oppression. But ultimately, if people are forced to leave their homes for reasons beyond their control and have no alternative, they are all refugees. The migration is the same; only the reasons differ.
Interviewer:
It is estimated that one billion people will become climate refugees within the next 50 years, according to UNESCO. What do you think about this?
Participant:
Human migration is natural, especially when there is necessity. However, if the world cannot manage this in a disciplined way, major chaos will occur. Without awareness and precautions, this could lead to wars and conflicts. We saw this during the COVID-19 pandemic—countries did not even help each other in times of crisis. In moments of desperation, moral values can easily be abandoned.
If humanity already foresees that climate change will create climate refugees in 30–50 years, then preventive measures must be taken. Emergency food and water aid systems should be prepared. However, when we talk about one billion people, it becomes questionable whether migration can be prevented or managed at all. Preventive and humanitarian measures should be prioritized.**Interviewer:**
Who do you think is responsible for the emergence of climate refugees?
Participant:
There are many reasons, but fundamentally, humanity consumes excessively. The world produces far more than it needs, yet resources are not shared equally. A small portion of the global population consumes the majority, while others suffer. If resources were shared more fairly, many of these problems could be reduced.
Interviewer:
How would you evaluate societal or political perspectives toward climate refugees?
Should they be viewed differently from other refugees?

Participant:
Political refugees are often viewed differently. Climate refugees, however, seem to be the
responsibility of all humanity. Climate change is the result of how we have exploited the
planet. Therefore, its consequences should be collectively addressed by all humanity.
Interviewer:
Do you think climate refugees should be granted citizenship?
Participant:
This is a very complex issue. If one billion people migrate, serious social chaos and even
wars could occur. Poorly managed policies would worsen the situation. Instead of permanent solutions like citizenship, rehabilitation and local solutions should be prioritized. If humanity learns to respect nature as a living entity, even if not completely,
this process can at least be slowed.
There is no clear yes-or-no answer to this issue. It is a gray area that requires scientific
and rational thinking.
Interviewer:
Do you think the concept of climate refugees should be taught in Social Studies courses?
Participant:
Absolutely. We are talking about the next 50 years. Today's children will be tomorrow's
decision-makers. They need awareness. This topic can be included as a subheading in the
curriculum. Teachers also need awareness. Even if it is not explicitly named "climate
refugee," it can still be addressed within climate-related topics.
Interviewer:
Have you ever covered this topic in your classes?
Participant:
We discussed climate issues in history classes, but not explicitly under the concept ofclimate refugees. However, it can definitely be introduced as a concept. Even if it is not
formally recognized today, it may be recognized in the future.
Interviewer:
refugees?
What teaching methods or materials would you recommend for teaching climate
Participant:

The most effective approach would be to present real-life stories and experiences of
climate refugees visually and emotionally. Empathy-based methods are important.
Interviews, documentaries, and visual evidence of environmental destruction should be
used to create lasting impressions.
Interviewer:
As a Social Studies teacher, what can be done to increase awareness?
Participant:
Universities can organize seminars, invite teachers, and conduct pilot studies in schools.
Teachers who are willing and informed can voluntarily raise awareness. The curriculum
already provides flexibility for this.
Interviewer:
Should climate refugees be educated differently from other refugees?
Participant:
They should be supported through cultural adaptation programs. If they are staying
temporarily, education should focus on integration. Many climate refugees come from
agricultural societies. With proper planning, they could be integrated into underutilized
agricultural areas through voluntary and well-managed programs.
Interviewer:
Is there anything else you would like to add?
Participant:
happy.
This is a very important and timely study. If I can contribute in any way, I would be
PARTIPICANT 6 (K6)
Interviewer:
Generally speaking, these individuals are not migrants. Migration is usually based on
choice. Migrants are individuals who move in order to improve their living standards.
However, if we distinguish the concept of refugees, refugees are individuals who no
longer have any choice. While migration aims to increase life comfort, refugees areindividuals who move in order to meet their basic needs for survival. Climate refugees, as

the name suggests, are displaced due to climate-related reasons. This concept is also
expressed differently, such as environmentally induced migrants or environmental
degradation–related migrants. However, the term *climate refugee* has become more
established in recent years. Although it is not legally recognized as a refugee status by the
United Nations, it is grounded in the 1948 Universal Declaration of Human Rights. The
United Nations classifies refugees based on certain criteria. Even though climate refugees
do not yet have a clear legal definition or status, there is extensive literature on this issue.
We are conducting this study for the first time in the field of education, so I wanted to
define it briefly.
Welcome, teacher.

Interviewer:
How many years have you been working as a teacher?

Participant:
I have been working for three years; by the end of this year, it will be three years.

Interviewer:
Your field is Social Studies, correct?

Participant:
Yes, Social Studies.

Interviewer:
Have you completed a PhD?

Participant:
Unfortunately, no.

Interviewer:
Hopefully in the future.

Participant:
not attempt it.
God willing. My main difficulty is language. Without sufficient language skills, I could

Interviewer:
Language is essential for academic progress.

Participant:
Exactly. Otherwise, one only completes degrees without advancing further.

Interviewer:
Shall we begin?**Participant:**
Yes, please.

Interviewer:
Have you ever encountered the concept of climate refugees before?
Participant:
Not under this name. I had heard of people migrating due to drought, but not described
explicitly as climate refugees.
Interviewer:
Was this in Türkiye or abroad?
Participant:
I have heard of people coming from Iraq and Syria. Syria is already very current. We also
see people migrating due to drought and lack of arable land from places like Mardin.
Some have even migrated further, toward Cyprus. Water scarcity has begun there as well.
However, this issue was never conceptualized clearly. Through your explanation, it has
now become clearer to me.
Interviewer:
Have you ever received training or attended a seminar on climate refugees?
Participant:
No. I have attended seminars on migration in general, but not specifically related to
climate.
Interviewer:
Have you seen any news reports explicitly using the term "climate refugee"?
Participant:
Unfortunately, no. As you know, the news we follow does not adequately reflect today's
real problems.
Interviewer:
Do you think there is a difference between climate refugees and other refugees?
Participant:
Yes. Climate refugees migrate out of necessity. War and blood feuds are different causes.
But with climate-related displacement, the land no longer sustains you. The soil itself
rejects you.
Interviewer:
UNESCO estimates that one billion people may become climate refugees within the next
50 years. What are your thoughts on this?
Participant:

Our village used to be rich in water. In the Mardin region, it was well known for its water
resources. Now it is becoming arid. The population has decreased from over 100 households to fewer than 50. This is due both to drought and lack of economic opportunities. The land no longer sustains people, and water resources are disappearing.
Interviewer:
So this is largely drought-related.
Participant:
Yes, drought is the main factor.
Interviewer:
Who do you think is responsible for the emergence of climate refugees?
Participant:
There is unconscious consumption. Especially water consumption. Traditional irrigation
methods are still used in agriculture, and nature is not protected. Forests are destroyed
under the assumption that they will grow back. We are accelerating climate change, and
now we are paying the price for it.
Interviewer:
Should climate refugees be considered a vulnerable group?
Participant:
Absolutely.
Interviewer:
How should society or politics approach climate refugees?
Participant:
It is difficult to evaluate them exactly like war refugees. There is no immediate threat like
bombs or guns. However, in the future, when billions are affected, perspectives may
change. These people also need to survive.
Interviewer:
Should climate refugees be granted citizenship?
Participant:
At present, it is very difficult. But in 20–30 years, maybe sooner, citizenship may become
inevitable. Hunger leaves no alternative.
Interviewer:
Do you think this topic should be taught in Social Studies courses?**Participant:**
Yes. We already discuss water conservation in the 5th grade, especially under the new
curriculum. This issue should not be limited to Social Studies; interdisciplinary

coordination would be beneficial because water will become one of humanity's greatest
problems.
Interviewer:
Have you ever taught this topic in your classes?
Participant:
Not under the title of climate refugees, but we discuss water scarcity, the future value of
water, and its societal impacts.
Interviewer:
Which teaching methods would be most effective?
Participant:
Field-based learning would be ideal, but it is very difficult in our country. Simulation or
imitation activities might help. Without experiencing hardship, children cannot truly
understand the value of water.
Interviewer:
What can Social Studies teachers do to raise public awareness?
Participant:
Projects supported by TÜBİTAK, seminars involving parents, nature trips, and camps
could be effective. Parents are key stakeholders. Living together with displaced people,
as we did with Syrian refugees, could also increase awareness, although this is socially
challenging.
Interviewer:
How should education be provided to climate refugees themselves?
Participant:
They do not need theoretical education; they already live the reality. Instead, practical
education should be provided—such as water-efficient agriculture, drip irrigation, and
alternative farming techniques. Countries like the Netherlands show what can be achieved with limited land and water. Education should be applied and locally adapted.
Interviewer:
Is there anything else you would like to add?
Participant:
Climate refugees will become one of the major problems of our time. This concept has
entered my vocabulary thanks to you. If precautions are not taken, demographic

structures will change, and social order may be disrupted. Water should be treated as anational priority. Lakes, rivers, and water resources in Türkiye are drying up rapidly, and
this affects the entire ecosystem. The state must take primary responsibility for this issue.

Interviewer:

Thank you very much.

Participant:

Thank you.

PARTIPICANT 7 (K7)

Interviewer:

Therefore, from a legal perspective, because migration is expected to increase, the concept is not yet widely recognized by the United Nations. However, when we examine
the core of the issue, climate refugees are individuals who are forced to leave their region
due to climate-related disruption. This is not a matter of choice. These individuals are
compelled to migrate and thus qualify as refugees.

That is the correct interpretation.

So, we have clarified the concept. Let us continue from there. How many years have you
been working as a teacher?

Participant:

This is my fifth year.

Interviewer:

Your fifth year. Then you are a graduate of Social Studies.

Participant:

Yes, I am a Social Studies graduate.

Interviewer:

What about postgraduate studies—have you completed a master's or PhD?

Participant:

No, I have not, but I am considering it.

Interviewer:

Hopefully at Yıldız University. We look forward to it.

Participant:

Hopefully.

Interviewer:

Have you ever encountered an individual who could be defined as a climate refugee?

Participant:

Well, as you know, throughout history people have not migrated due to a single reason

alone. I think climate is sometimes one of the main contributing factors rather than the sole cause. This applies both to international refugees and internal migration, although the latter is a separate issue. I have not encountered someone who could be clearly defined as a climate refugee, but I have encountered people for whom climate was one of the contributing reasons for migration.

Interviewer:

Were these migrations domestic or international?

Participant:

International. For example, people coming from Syria or Iraq, from the southern regions. Their concerns are not only war, uncertainty, or economic conditions. In some cases, even if it is a minor factor, climate-related reasons also play a role. Over time, the construction of dams, gradual climate change, the inability to grow certain crops, and water scarcity have also caused people to migrate here.

Interviewer:

Let me briefly explain why I became interested in this topic. While talking with a colleague, I heard about a family coming from Syria whose migration was not solely war-related. A teacher friend mentioned that they migrated due to a conflict with neighbors over water usage in their apartment building. After hearing this, I became more interested in the issue. Later, while writing my thesis, I encountered similar discussions in the literature and connected the concepts. This topic has not been studied much in education, and its definition is quite challenging. Encountering someone who explicitly identifies as a “climate refugee” is also difficult, as the concept is not legally recognized and thus has limited presence in the literature. I wanted to share this process with you because I experienced similar challenges while exploring the concept.

How would you define a climate refugee?

Participant:

A climate refugee is someone who cannot sustain life in their place of residence due to

living conditions or economic difficulties and therefore migrates to another place to
survive better and improve their quality of life.
Interviewer:
Thank you. Have you ever received training or seen news related to climate refugees?
Participant:
No. We usually address climate change in Social Studies under contemporary world
problems, such as global warming. However, this topic is treated as a subtopic, and since
it is not clearly defined, I have not encountered it explicitly.**Interviewer:**
Do you think there is a difference between climate refugees and other refugees?
Participant:
In my view, survival comes before quality of life. Therefore, refugees fleeing war are the
most urgent group, as their lives and security are immediately threatened. It is natural for
countries to prioritize war refugees because their situation is more urgent and life-threatening.
Interviewer:
UNESCO estimates that one billion people may become climate refugees within the next
50 years. What are your thoughts on this?
Participant:
Life is constantly changing, and I believe such developments will occur. These changes
will inevitably bring difficulties and new concepts into the literature. It is important to
address these issues early, research highly probable scenarios, and propose solutions in
advance.
Interviewer:
Can climate refugees be defined as a vulnerable group? To what extent?
Participant:
Any factor that reduces a person's quality of life creates vulnerability. However, is it an
immediate vulnerability? Not as urgent as war-related displacement. Still, in the long
term, it is a serious issue. It is a negative situation, and these people need to be
understood and supported with appropriate solutions.
Interviewer:

Who do you think is responsible for the emergence of climate refugees—individuals,
governments, institutions?
Participant:
Primarily, I think the most influential institution should be the United Nations. This is not
the problem of a single country. If people are migrating from one place, they must migrate to another. Every country has the potential to receive refugees, and every country
affected by climate change risks losing its population. Therefore, this is a global issue
affecting the entire world.
Interviewer:
Should climate refugees be granted citizenship?
Participant:
Although the United Nations provides certain recommendations, citizenship laws are
determined by each country's internal legal structure. Therefore, practices differ worldwide. Whether citizenship should be granted depends on the views of the citizens ofthe host country. If a country is to remain livable, the welfare of its citizens should not be
reduced. In democratic systems, a referendum could be held to reflect public opinion.
Interviewer:
Should this topic be taught within the Social Studies curriculum?
Participant:
Absolutely. It may not be possible to allocate an entire course to it due to time constraints, but it should certainly be included as a unit or topic. Climate is fundamental—it is our living space, our source of food, and the foundation of life. The
consequences of environmental degradation must be addressed in textbooks.
Interviewer:
Have you ever taught this topic? Which methods would you use?
Participant:
I address climate-related topics such as global warming, agriculture, and environmental
issues, even if I do not explicitly use the term "climate refugee." These topics are already
present in textbooks. As for methods, traditional teaching can be effective, but project-
based activities can also be used. For example, awareness-raising theater activities could

be organized. Students could prepare a play on this topic and perform it for the entire
school, which would help students become more familiar with the concept.
Interviewer:
As a Social Studies teacher, what can be done to raise awareness at the societal level?
Participant:
Since we live in a digital age, television, media, and the internet should be utilized. Public service announcements supported by the state could be broadcast to raise awareness among large audiences.
Interviewer:
If education were to be provided to climate refugees, what kind of education should it be?
Would it differ from that given to other refugees?
Participant:
Education should focus on how to cope with environmental challenges rather than simply
escaping them. For instance, if there is drought, people often migrate because they lack
the resources or economic capacity to access water. Training on sustainable water use,
alternative agricultural practices, and adaptation strategies could help prevent forced
migration.
Interviewer:
Is there anything else you would like to add?**Participant:**
No, that would be all. Thank you very much.
Interviewer:
Thank you for your time and contributions.
Participant:
Thank you. I appreciate your effort. Best of luck.
PARTIPICANT 8 (K8)
Let me give some background information first.
What we call *climate refugees* are people who are forced to migrate from their place of
residence due to climate-related adverse conditions such as extreme weather events,
desertification, drought, floods, or sea-level rise. In other words, these individuals are
compelled to leave their regions because of climate-related reasons.
It is important to distinguish between the concepts of *refugees* and *migrants*. I realized

this clearly while talking with a friend the other day. Migrants generally move in search
of better living conditions, whereas refugees migrate due to compulsory and unavoidable
circumstances. Therefore, climate refugees are people who are forced to migrate from
their regions specifically due to climate-related factors, often to different regions or
countries.
Although many of these movements currently occur within national borders, climate
refugees have increasingly become a group that creates problems connected to international borders and other countries. According to last year's data, there were approximately 9.8 million climate migrants, within a total of 184 million migrants worldwide. This is a term that is still not widely known or recognized by many people.
There is also growing concern that the number of climate refugees will increase significantly in the coming years.
After providing this information, I would like to give some personal details. I am female,
I have two years of teaching experience, and I graduated from a social studies education
program. I work at an Imam Hatip secondary school, which is classified as a project
school.

Does your school have any environmental focus or sensitivity related to environmental issues?

No, not at all.
None.
Zero.
I tried to work on this, but it did not work out.Actually, we have very beautiful areas within our school that could be described as
forested land. However, unfortunately, these areas are very neglected. We even have
animal shelters and take care of animals at school, but the forested areas are not maintained at all. Neither the school administration nor the service staff take care of
them. They have been left completely idle. Occasionally, the municipality comes, cuts
some branches of the trees, and leaves.
They only make superficial arrangements.
I would not call it superficial exactly, but our trees are very large. They only cut a few

branches so that the school entrance is not blocked, and then they leave.
So nature is seen merely as an obstacle.
Yes, exactly. Completely so.
Have you ever encountered a climate refugee before?
No, I haven’t. There are many refugees at our school, but they are not related to climate issues. They usually came because of war or in order to receive better education.
After this explanation, how would you define a climate refugee?
I heard this term for the first time here.
But after learning the concept, I would define a climate refugee as someone who is forced to migrate due to climate-related adverse conditions and the deterioration of living conditions.
Have you ever received any training or attended a seminar on climate refugees before? Is there a difference between climate refugees and other refugees?
There actually is a difference. I learned about climate refugees only now. Previously, when I thought of refugees, I only associated them with war or escaping from political oppression. Now that I have learned about climate-related migration, I think there is a major difference. In wars, people may be able to return when conditions improve. But since climate conditions may not improve and are actually worsening, climate refugees may not be able to return to their homelands.
What do you think about climate change causing forced migration? There is data suggesting that nearly one billion people may be affected within the next 50 years.
That seems possible. Even when we consider our own country, for example the Konya region, it is geographically large but has a decreasing population and experiences out-migration, as we explain in textbooks. With increasing drought in Central Asia, people migrate to our country or to European countries. However, I am not sure what measuresare taken or what policies exist regarding this issue. I do not think there is sufficient awareness or concrete action.
Can climate refugees be defined as a disadvantaged group?

Yes. They are forced to leave their homeland and move to another place where they must
be socially accepted. Being disconnected from one's homeland and culture makes adaptation very difficult. After all, refugees do not migrate by choice. They are forced to
do so. Ideally, their living conditions should have been improved where they originally
lived.

Who do you think is responsible for the emergence of climate refugees?

States and political leaders. Actually, all states. Ultimately, responsibility lies with governments and societies as a whole.

How do you evaluate the social or political perspectives toward climate refugees?
Should they be granted citizenship?

This is a bit political. From a humanitarian perspective, citizenship should be granted.
However, the issue of registration and verification is important. How can we be sure that
people are truly migrating due to climate reasons? Similar debates exist regarding refugees from Syria. Not everyone who arrived may have been directly affected by war.
For example, I do not currently see active war in Afghanistan. Our country's borders are
open to everyone. If proper monitoring and regulation can be ensured, then citizenship
should be granted on humanitarian grounds.

Do you think climate refugees should be taught within the social studies curriculum?

Absolutely. Even when we teach climate topics in social studies textbooks, they are
covered very superficially. Teaching students about environmental awareness and nature
conservation would be very beneficial. Migration is already a topic, and climate is
another topic. Climate refugees could connect both themes very well. Integrating this
topic into the curriculum would be much more effective.

Which methods or techniques should be used to teach students about climate refugees?

Students should learn through experience—by doing and living. If they can somehow
confront these conditions, even through videos or simulations, and put themselves in the

place of climate refugees, learning would be more meaningful.
So, empathy-based learning?
Yes, exactly.**As a social studies teacher, what can be done to raise awareness about climate refugees?**
Conferences can be organized. Teachers should also be educated first. I was not aware of this topic myself. Even if it is not included in textbooks, we can still talk about it during class, even if only for the last five or ten minutes. If teachers become more aware, they can pass this knowledge on to students. Children do not encounter this topic in the media either, so if we do not teach it, they will not learn it elsewhere.
What should be prioritized when providing education to climate refugee students?
First and foremost, language education. To be accepted and to communicate in a new country, language proficiency is essential. Other aspects can follow gradually, but language should come first.
PARTIPICANT 9 (K9)
My main purpose is to examine social studies teachers' views on climate refugees.
Since you are a relevant and accessible participant, I wanted to ask for your support and gather your opinions.
First, I would like to explain the concept of *climate refugees*. Climate refugees are individuals who are forced to leave their place of residence due to adverse conditions caused by climate change. These individuals migrate in search of a safer place because of environmental threats such as floods, drought, desertification, and sea-level rise. The difficulties they experience during displacement clearly reveal the human dimension of the climate crisis.
Normally, it should be noted that there is no formal legal definition of "climate refugee" recognized by the United Nations or the European human rights framework. However, in the academic literature, this group is increasingly referred to as *climate refugees*, and sometimes also as *environmental migrants*. In this study, we use the term *climate*

refugees to refer to individuals who are forced to leave their living areas due to climate change–related conditions.
Based on this definition, we will continue our discussion.
How many years have you been teaching, Serpil?
I have been teaching for eight years.
Your field of graduation is social studies, correct?
Yes, social studies.
What type of school do you work at?
A middle school.**Have you ever encountered a climate refugee before?**
No, I haven't.
After hearing this explanation, does the concept make sense to you?
Yes. Actually, this is a concept that I encountered mainly through your study. It is new to me as a term, but I realized that although I did not know the concept by name, I was already familiar with the phenomenon itself. Climate change and the resulting deterioration of living conditions forcing people to migrate is something we are aware of.
So, even if I did not know the term "climate refugee," the situation itself was not unfamiliar.
In your opinion, is there a difference between climate refugees and other refugees?
If so, what are the differences?
Compared to other refugees, such as those fleeing war or major political conflicts, the causes of migration are more easily recognized because the reasons are clearer. In the case of climate refugees, the causes are not as immediately visible or direct, which makes them harder to recognize. Climate refugees are also forced to migrate, just like other refugees, but because this process occurs over a longer period of time, it is less noticeable. While war-related migration is often associated with immediate threats to life, climate-related migration unfolds gradually. Despite these differences, both groups experience forced displacement.
What are your thoughts on climate change causing forced migration, particularly in relation to international organizations?
Climate change affects many areas, especially agriculture and water resources. Countries

located in certain geographical regions—particularly small or medium-sized countries—
are likely to be more affected by these changes. Over time, this will inevitably lead to
migration movements, possibly toward northern regions. However, unlike other refugee
movements, these migrations may not occur suddenly or in large groups but rather gradually.

Do you consider climate refugees to be a disadvantaged or victimized group?

Yes, I have thought about this. In cases such as civil wars, certain groups within a
country may benefit while others suffer more. However, climate change affects an entire
country rather than a single group. Still, lower-income groups, rural populations, and
people whose livelihoods depend directly on nature and agriculture are likely to be more
severely affected. Therefore, while climate change impacts everyone, vulnerable groups
experience its consequences more deeply.

Who do you think is responsible for the increase in the number of climate refugees?

Government policies play a major role. State authorities and decision-makers bear significant responsibility in preventing or mitigating these outcomes. However, this is
also a collective responsibility. While governments have a greater role, individuals also
share responsibility. Still, I believe the primary responsibility lies with state authorities.**How do you evaluate societal perspectives toward climate refugees? Should they be
granted citizenship?**

This is a challenging issue, and I cannot approach it very optimistically. Granting citizenship to climate refugees could lead to serious debates. However, if people are
forced to migrate because their living areas have become uninhabitable—such as due to
sea-level rise or severe environmental degradation—then this situation should be evaluated differently from voluntary migration. If people have no possibility of survival
in their homeland, then offering citizenship could be considered. In cases where life itself
is at stake, such an option becomes more justifiable.

Do you think the concept of climate refugees should be included in the social studies curriculum?

Yes. Although this concept is new to me, when I consider the learning outcomes of social studies, I believe it is a necessary concept. We already address related issues implicitly, but including it explicitly as a concept would draw more attention and increase awareness.

If you were to teach this topic, which teaching methods or techniques would you use?

This would depend on the students' level and the context, but I think using case studies would be very effective. There are many real-world examples from around the world that could be used. Reaching conclusions through real-life cases would help students understand the issue more deeply.

What can be done in social studies classes to raise awareness about climate refugees?

Students should be encouraged to conduct their own research. When students investigate problems themselves or prepare presentations, learning becomes more meaningful. Involving students actively in the process would make education more effective.

If you were teaching a climate refugee student, which methods would you use in your classroom?

Since the student has lived through the experience, listening to and sharing their experiences would be very valuable. Activities such as role-playing or drama could be used to help other students empathize. These methods would make the student more active in the learning process and help build understanding among classmates.

Is there anything else you would like to add?

No. I think this is a very valuable topic and one that could contribute meaningfully to social studies education. I hope it finds a place in the new social studies curriculum

PARTIPICANT 10 (K10)

At the beginning of this interview, I provided general information about the concept of climate refugees.

As you may have noticed, climate refugees refer to individuals who are forced to migratedue to climate-related reasons. In other words, these people are not migrants by choice;
they are refugees. Their numbers increase every year. According to 2024 data reported by
UNESCO, out of approximately 84 million displaced people worldwide, 9.8 million are
classified as climate refugees. This situation has now become clearly established in the
data. That is why I wanted to focus on this topic.

Have you ever encountered a climate refugee before?

To be honest, I have not directly encountered a climate refugee. However, I have come
across indirect cases. For example, floods, drought, and desertification are major problems. What do these lead to? Economic decline. This, in turn, leads to job losses. As
a result, people are forced to migrate due to loss of livelihood. For instance, floods can be
very decisive. Someone loses their shop, job, or home because of a flood or a landslide.
These types of disasters are increasing, and they lead to internal migration between cities.
This can also cause population redistribution within a country.
There are also international examples, particularly from Southeast Asia. Countries such
as Myanmar, Malaysia, or regions around Bhutan experience frequent climate-related
disasters. These countries already face economic difficulties, so people tend to migrate to
countries closer to the West, such as Türkiye, hoping to sustain themselves economically.
Additionally, migration from southeastern Türkiye can also be considered climate-
related. Although this is not always explicitly recognized, it still qualifies as climate
migration. Projects like GAP have attempted to address this issue, but migration continues. There are many examples of climate-related migration within Türkiye. For example, in the Konya region, increasing drought has caused significant damage to
agricultural land. Sinkholes formed due to groundwater depletion have destroyed farmlands. Farmers who once cultivated hundreds of acres can no longer sustain agriculture. As a result, they are forced to abandon farming and migrate to cities, often

seeking factory jobs. This leads to increased urban migration.
In southeastern regions such as Mardin, Şanlıurfa, and Şırnak, desertification and drought
are severe. Although Konya and Karapınar are often highlighted, these southeastern
regions are also experiencing intense environmental degradation, which drives migration.

How would you personally define a climate refugee, apart from the academic definition?

In my view, a climate refugee is someone who is forced to leave their place of residence
entirely due to natural factors. Once a person is compelled to leave for survival reasons,
they become a refugee wherever they go. There is a necessity involved—people must
earn their living. If someone cannot survive through farming due to climate conditions,
they will inevitably migrate, perhaps to a city like Istanbul, to work in a factory. This is a
matter of survival.**Have you ever received any training or education related to climate refugees, or
encountered this topic in the media?**

I am familiar with the concept through academic literature and news coverage, but I have
never received formal training on this topic. I know the concept, but not through
institutional education.
In fact, I would argue that if you interviewed 200 teachers, maybe only two or three
would say they know what a climate refugee is. Among the six interviews you have
conducted so far, none of the participants were familiar with the term. Even district-level
or school-level subject coordinators are unlikely to know this concept unless they have
engaged in specialized academic reading.

**Do you think there is a difference between climate refugees and other types of
refugees?**

Absolutely. For example, political refugees may leave their country due to ideological
conflicts or political persecution. Some may even be fleeing legal consequences. In

contrast, climate refugees cannot be categorized based on political, religious, ideological, or ethnic grounds. Their situation is purely based on environmental necessity. No one can reasonably object to their displacement; they are victims. Therefore, climate refugees should be considered a primary category among refugee groups, as their displacement is directly tied to survival needs.

Of course, humanitarian crises in places such as Palestine, Iraq, and Syria involve extreme suffering and should not be diminished. However, these situations fall outside the specific scope of climate-induced displacement that we are discussing here.

Who do you think is responsible for the increase in the number of climate refugees?

Primarily, political decision-makers. This includes ministries such as Education, Health, and Environment and Urbanization. In countries like Türkiye, decisions are made at the top and implemented across society. Without strong public awareness and political will, individual efforts alone are insufficient. Once a solid foundation is established through policy, people will follow, just as they comply with public health regulations.

Climate change is expected to displace nearly one billion people over the next 50 years. What are your thoughts on this?

This is a global problem that will affect all countries, including developed nations such as the United States. Climate-induced displacement is not limited to war zones. Therefore, both public awareness and political action must be strengthened to address this challenge.

Should climate refugees be granted citizenship?I believe there must first be a proper evaluation process to ensure that integration will not cause social problems. Even refugees fleeing war undergo screening in Western countries. Accepting people without regulation can lead to challenges, as Türkiye has already experienced. Therefore, migration management centers should conduct thorough assessments.

One reason climate refugees are not formally recognized is that states fear an increase in

numbers and the economic burden this may create. This is also why the concept has not
yet been legally defined by international institutions. While it is widely discussed in
academic literature, it lacks legal recognition.

Do you think the concept of climate refugees should be included in the social studies curriculum?

Without question. This concept must be included in social studies textbooks and curricula. When teaching globalization or climate change, failing to address its impact on
migration leaves the topic incomplete. Students can easily engage with and discuss this
concept.

Have you ever taught this topic in your classes?

Yes, indirectly. For example, we discussed the major wildfires in Australia three years
ago and the resulting migration toward Indonesia and New Zealand. Although we did not
explicitly use the term "climate refugee," we addressed forced migration due to natural
disasters.

Which teaching methods would be effective for this topic?

Outdoor learning environments are crucial. Taking students to forested areas and then
showing desert environments through virtual museums creates powerful comparisons.
This helps students understand economic and environmental disparities. Empathy-based
activities are also important—for example, asking students to imagine losing their
livelihood due to climate-related disasters.
I have personally taken students to areas where water resources have dried up. When
students directly observe these conditions, learning becomes far more meaningful.
Experiential learning is more effective than abstract instruction.

What can social studies teachers do to raise awareness about climate refugees?

This topic must be integrated into the national curriculum and platforms such as EBA.
NGOs like TEMA or the Red Crescent should be involved in schools so students can
interact directly with experts. Seminars and structured educational content should be

developed.
Should education for climate refugees differ from that of other refugees?Yes. Orientation programs are essential. Climate refugees need guidance on employment
opportunities, adaptation, and psychological support. Rehabilitation and integration
support must be prioritized.
Is there anything else you would like to add?
No, I think we covered everything sufficiently.
PARTIPICANT 11 (K11)
Problems may arise from drought, rainfall, flooding, or similar environmental issues.
Especially in some island regions, settlements are becoming uninhabitable due to sea-
level rise. Migration may occur as a result of rising sea levels. In some regions, destruction caused by extreme typhoons and strong winds can also lead to displacement.
However, more commonly, this migration is caused by drought and desertification. In
Southeast Asia, migration increasingly occurs due to rising sea levels or excessive rainfall, which damages forests and ecosystems. These environmentally driven migrations are what we actually refer to as **climate refugees**.
At this point, it is important to clarify something: climate refugees are not people who
migrate simply because the climate is more comfortable elsewhere—for example, moving from a hot city like Adana to a cooler region such as Trabzon for summer housing. That is not the case. For instance, Adana is very hot, and some people from
Kahramanmaraş have moved there, but those people are actually internal migrants.
Similarly, someone who dislikes their country's living conditions and moves to Canada is
called a migrant. Those who flee political pressure are defined as refugees or asylum
seekers.
However, individuals we call climate refugees are not people who move because of
discomfort or reduced comfort levels. They are individuals with real survival concerns.
They migrate because living conditions no longer allow them to survive. Therefore, this
concept must be clearly defined. In Türkiye, it is not yet a well-known concept and has

not been studied extensively in academic literature. In fact, this is the first time I am
trying to work on it. That is why it needs to be explained in detail.
The reasons are, of course, economic as well. Beyond that, according to **UNESCO** and
UNEP, projections indicate that over the next fifty years, climate-related migration could
affect up to one billion people, meaning a massive refugee population. That is why this is
a long-term issue we are working on.
I am female. I have been working for seven years now.
Yes, exactly. I have been in my seventh year of service. My subject area is social studies.
Previously, I worked in Cizre.I worked at Menderes Middle School. It was a regular middle school, not an Imam Hatip
school.
Currently, I work at Vakıflar Middle School. It is also a regular public school, not an
Imam Hatip school.
When we look at the school's identity and its relationship with environmental issues,
environmental education is not strongly emphasized. The school is located in the Bağlar
area, and the socio-economic level is relatively low, but overall the student profile is
average.
Thank you.
Have you ever encountered a climate refugee before?
No, I have not.
If you were to define a climate refugee based on life experiences, how would you describe it?
A climate refugee is a person who is forced to leave their place of residence due to
extraordinary climatic conditions.
Thank you.
Have you ever received any education or training related to climate refugees?
No, I have not.
Have you seen anything about climate refugees in the media?
No, I have not.
Climate refugees are forced to leave without hope of return. Other refugees may return to
their countries when wars end or conflicts are resolved, but since the climate is unlikely

to improve, climate refugees face a more permanent displacement.
What do you think about the claim that climate change will affect one billion people
within the next 50 years?
Considering that the climate is becoming increasingly unstable, with more extreme heat
and cold, and that seasonal transitions have weakened, more drought may occur due to
global warming. As a result, more people may be forced to migrate. This situation would
affect not only the economy but also social life and even the cultural structures of other
countries.
Could Turkish citizens also be considered within this scope?I think they are a highly vulnerable group. When I worked in Cizre, the heat was extreme.
During summer months, people could barely go outside. Production stops during certain
hours of the day and certain months of the year. This affects both the economy and
people’s daily lives. I believe this is a very serious form of hardship.
Who do you think is responsible for the emergence of climate refugees?
I am not sure whether precautions can truly be taken against climate change, but perhaps
when borders were drawn, geography played a determining role—“geography is destiny,”
as they say.
Have you ever had any experience regarding social or political perspectives toward
climate refugees?
I have not personally experienced this, but I believe these people should be supported
because they are displaced for reasons beyond their control. Since climate cannot be
changed by individual effort, they cannot simply be told to work harder. Therefore,
assistance should be provided, and migration to other countries should perhaps be tolerated.
Do you think climate refugees should be granted citizenship?
I think they should. Because they can no longer use the natural resources of their environment due to climate conditions.
Social studies focuses on current issues, and this is a current—and even overlooked—

problem. If climate refugees’ daily lives and integration into society are discussed in lessons, and students become more aware, they may contribute to climate solutions in the future.

Which methods and techniques would you use to teach this topic?

Geography should definitely be utilized. Field studies, environmental observations, and trips could be organized. Students could be taught how to transform climatic challenges into opportunities—for example, using solar energy in very hot regions or wind energy in storm-prone areas. This would raise awareness about converting negative conditions into positive outcomes.

As a social studies teacher, how would you raise awareness about climate refugees?

We could show documentaries about climate-related suffering. Afterward, we could use discovery learning, brainstorming techniques, or project-based learning. Students could be asked how they would act or what kind of projects they would develop if they were in such situations.When educating climate refugees, how much attention should be given compared to other migrants?

If there are still resources that can be developed in their home countries despite climate challenges, and if climate conditions can be transformed into energy or production, I would support education aimed at helping them remain and develop their regions.

However, if nothing can be done and survival is no longer possible, then integration into the host society should be supported.

Thank you very much.

Is there anything else you would like to add?

No.

Thank you.
