# Supplementary material for: Understanding climate refugees from educators’ perspectives: Social studies teachers’ views
Source: PLoS One. 2026 Mar 16;21(3):e0344777. doi: 10.1371/journal.pone.0344777 (PMC12991243; doi:10.1371/journal.pone.0344777)
Supplement: S1 Table — (PDF) [file pone.0344777.s002.pdf]

Table 1. *Distribution of Teachers by City, Duration of Occupation, Education Level and Gender*

**Participant**	**City**	**Department**	**Year**	**Education Level**	**Gender Male/ Female**	**Interview Duration**
K1	Karaman	Social Studies Teacher Education	20	PhD	M	26
K2	Istanbul	Social Studies Teacher Education	2	License	M	16
K3	Malatya	Social Studies Teacher Education	3	Master	M	14
K4	Konya	Social Studies Teacher Education	12	License	F	17
K5	Istanbul	History Teaching	30	License	M	31
K6	Mardin	Social Studies Teacher Education	2	License	M	18
K7	Istanbul	Social Studies Teacher Education	6	License	M	16
K8	Ankara	Social Studies Teacher Education	3	License	F	15
K9	Kocaeli	Social Studies Teacher Education	7	License	F	14
K10	Istanbul	Social Studies Teacher Education	12	PhD	M	20
K11	Şırnak	Social Studies Teacher Education	8	Master	F	18
			**Total Duration**			204 minutes.
