# Supplementary material for: Understanding climate refugees from educators’ perspectives: Social studies teachers’ views
Source: PLoS One. 2026 Mar 16;21(3):e0344777. doi: 10.1371/journal.pone.0344777 (PMC12991243; doi:10.1371/journal.pone.0344777)
Supplement: Attachment — Submitted filename: Response to Reviewers.docx [file pone.0344777.s004.docx]

**Response to Reviewers**

**Page 4:** “While forming the study group of the research, diversity was increased with teachers of different academic levels and age groups working in provinces that have the potential to be seriously negatively affected by climate refugees”

**The statement about being seriously negatively affected by climate migration should be changed.**

The city may have been affected, but the teacher may not have been. Is there any evidence in the literature showing that these cities have been negatively affected by this situation?

**Page 4:** **The duration of the interviews must be specified.**

Were the meeting times rounded?

26 minutes, 24 minutes. No seconds?

**Page 6:** In this part of the research, the themes, sub- themes and codes are tabulated and presented with direct quotations from the participant views on each code.

Themes, sub-themes, and codes are presented in a table, but I couldn't see a table or figure.

A figure showing all themes should be provided, with the codes explained one by one below.

**Findings:** You should provide the explanation you gave in the last paragraph of each theme as a finding at the beginning of the theme. You should explain the teacher's opinions below.
